# Potential Local Mechanisms for Exercise-Induced Hypoalgesia in Response to Blood Flow Restriction Training

**DOI:** 10.7759/cureus.43219

**Published:** 2023-08-09

**Authors:** Giovanni A Cervini, Matthew Rice, Jeffrey L Jasperse

**Affiliations:** 1 Biomedical Sciences, Liberty University College of Osteopathic Medicine, Lynchburg, USA

**Keywords:** endcannabinoids, immune modulation, blood flow restriction training, mrgpr, exercise induced hypoalgesia

## Abstract

Overall, there is a great need within sports medicine to ensure that athletes can return from injury in an efficient, yet thorough manner. It is crucial to not avoid necessary difficulties in this process but also to ensure time-efficient rehabilitation. One of the more promising techniques to achieve timely recovery is blood flow restriction (BFR) training. BFR training is a growing and novel development that could be a vital tool to lighten the burden of recovery from injury in athletes. BFR utilizes a pneumatic tourniquet to limit blood flow in specific areas of the body. The use of BFR has been shown to potentially enhance the analgesic effects of exercise-induced hypoalgesia (EIH). By limiting pain, athletes will be less burdened by mobility and loading exercises required for them to effectively return to play. In a field where time away from sports can have massive implications, the need for tools to assist in the acceleration of the rehabilitation process is vital. Much of the work that has already been done in the field has been able to exploit the benefits of EIH and further enhance the body’s capabilities through BFR. Studies have compared EIH at low- and high-intensity settings utilizing BFR with both resistance and aerobic exercise. The results of these studies show comparable beta-endorphin levels with high-intensity exercise without BFR and low-intensity exercise with BFR. Low-intensity training with BFR had greater local pain relief, perhaps indicating the promising effects of BFR in enhancing EIH. By reviewing the current literature on this topic, we hope that further progress can be made to better understand the mechanism behind BFR and its ability to enhance EIH. Currently, local metabolites are a major focus for the potential mechanism behind these effects. Mas-related G-protein-coupled receptors (Mrgprs) contribute to local pain pathways via mast cell degranulation. Similarly, chemokine receptor 2/chemokine ligand 2 (CCR2/CCL2) triggers mast cell degranulation and inflammation-induced pain. Finally, pain-reducing effects have been linked to anti-inflammatory IL-10 signaling and anaerobic metabolites via transient receptor potential vanilloid 1 (TRPV1). Through a better understanding of these metabolites and their mechanisms, it is possible to further exploit the use of BFR to not only serve athletes recovering from injury but also apply this information to better serve all patients.

## Introduction and background

Injury and the burden of recovery

In the world of sports, an injury can be detrimental to the development of an athlete. When examining the numerous injuries athletes may endure, there seems to be a particular burden placed on the lower extremity of the musculoskeletal system. In professional soccer, for example, an athlete will be injured an average of two times per season [1]. The most common of these injuries are lower extremity muscle strain, ligament sprain, and contusion [1]. Likewise, when examining young athletes, the most common presenting injuries to emergency rooms include overuse injuries, acute traumatic knee injuries, and ankle sprains [2].

With the number of hours that athletes spend in training, the repetitive stress placed on the musculoskeletal system places athletes at high risk of injuries that have the potential to take athletes out of their respective sports for an extended period. In a professional and collegiate athletic setting, these injuries are of particular concern, as time away from a sport has the potential to significantly set back an athlete’s development in their respective sport. Considering that muscle regeneration peaks at two weeks after injury [3], even a minor injury, such as a muscle strain, has the potential to decrease the progression of an athlete significantly.

To treat these nagging injuries efficiently, the athlete will often undergo an intense training regimen to rebuild muscle, often guided by the principles of mobilization and loading. Essentially, these principles include various stretching and weight training techniques to strengthen the injured region [4,5]. A crucial development for athletes facing musculoskeletal injuries is the discovery that exercise-induced hypoalgesia (EIH) is enhanced through blood flow restriction (BFR) training. With most injury recovery regimens requiring some form of exercise-related resistance training, BFR could accelerate athlete recovery times through a reduction in pain associated with recovery.

BFR and EIH

EIH is a naturally occurring pain reduction that has been observed in individuals both during and following exercise [6]. In previous studies, it has been noted that much of the analgesic effect experienced through exercise depends on intensity. One must perform a continuous exercise session at 70% VO2 max for the body to release the beta-endorphins needed for pain reduction [7]. Other research [8] has demonstrated that high-intensity exercise for a short duration induces hypoalgesic effects, observing pain reduction at 85% VO2 max. However, when an athlete is physically unable to withstand heavy loads, achieving the workloads for recovery may not be possible. A potential mechanism to combat this hindrance is BFR training. This technique involves the partial or total occlusion of arterial and venous arterial flow in an exercising limb by pneumatic tourniquet [9]. Recent reports [9] in this area have demonstrated that similar increases in beta-endorphin levels can be observed in 40% VO2 max low-intensity exercise with BFR (11% increase) and 80% VO2 max high-intensity exercise without BFR (14% increase). Overall, the low-intensity exercise with BFR had a greater local hypoalgesic effect, and a comparable systemic hypoalgesic effect. Not only has BFR been demonstrated to improve hypoalgesia following aerobic exercise, but similar results have been demonstrated in resistance training as well [10]. Overall, beta-endorphin levels increased by 21% and 23% in low and high-pressure BFR resistance exercises, respectively. Low-pressure BFR corresponds to 40% limb occlusion pressure (LOP) and high-pressure BFR to 80% LOP. Due to these significant increases in neurotransmitters, individuals experienced hypoalgesia effects for as long as 24 hours [10].

Moreover, the analgesic effects of exercise extend outside the exercising limb, thus inhibiting nociception throughout the body [11]. From performing exercise, it has been noted that there is both a local and systemic change in pain. Exercise reduces pain and is thought to be related to the body’s naturally occurring production of opioids and endocannabinoids. Medical professionals across various specialties, including sports medicine, may be able to better treat their patients by incorporating BFR training into the already established rehabilitation regimens. BFR offers the option for reducing exercise intensity while maintaining comparable increases in muscle hypertrophy, strength, and pain reduction [12]. While the protocols may vary, the results of BFR training are consistent in demonstrating increased bone formation, lactate concentration, analgesia, and muscular hypertrophy [13]. Clinically, BFR training has repeatedly been tested and shown to be useful for injured patients experiencing pain and difficulties with rehabilitation in the lower extremities. A meta-analysis examining pain and strength found a reduction in pain (standardized mean difference, -0.61) and increasing strength (standardized mean difference, 0.83) following low-load resistance training with BFR [14]. Immediate pain reductions (*P* < 0.003) were seen with 80% LOP BFR training after various lower extremity exercises [15]. Furthermore, BFR with 50% LOP was demonstrated to be effective several years after an earlier anterior cruciate ligament repair; rectus femoris thickness, vastus lateralis thickness, and knee extensor strength increased by 11% ± 5%, 10% ± 6%, and 20% ± 14% (*P* < 0.01), respectively [16]. What remains unclear to date is the exact mechanism by which BFR augments the effects of exercise.

## Review

Potential local mechanisms of hypoalgesia during BFR training

Endocannabinoids Known to Induce EIH

EIH has been suggested by some to result from increased 2-arachidonoylglycerol, an endocannabinoid agonist [17]. However, the same study considered the possibility that there may be other underlying mechanisms. Hughes and Patterson [10] addressed this factor when noting an elevated pressure pain threshold (PPT) 24 hours after the initial BFR training session. PPT is the required amount of pressure applied to a testing region that would elicit a pain response. Similarly, Hughes and Patterson [10] examined the opioid and endocannabinoid systems. Their findings of no difference in the levels of 2-arachidonoylglycerol and normal beta-endorphin levels 24 hours post-BFR training suggest an alternative mechanism for EIH. In addition, there was a greater persistence in EIH in the exercising limb with limited systemic effects. Thus, it is possible that a local pain modulatory mechanism may be at play. Given the use of a tourniquet in BFR training, one potential explanation is the LOP may impede the outflow of a local factor, which would otherwise be washed out of the active muscle group via venous outflow. This factor is yet to be discovered.

Mast Cells and Mrgprs

**Figure 1:**
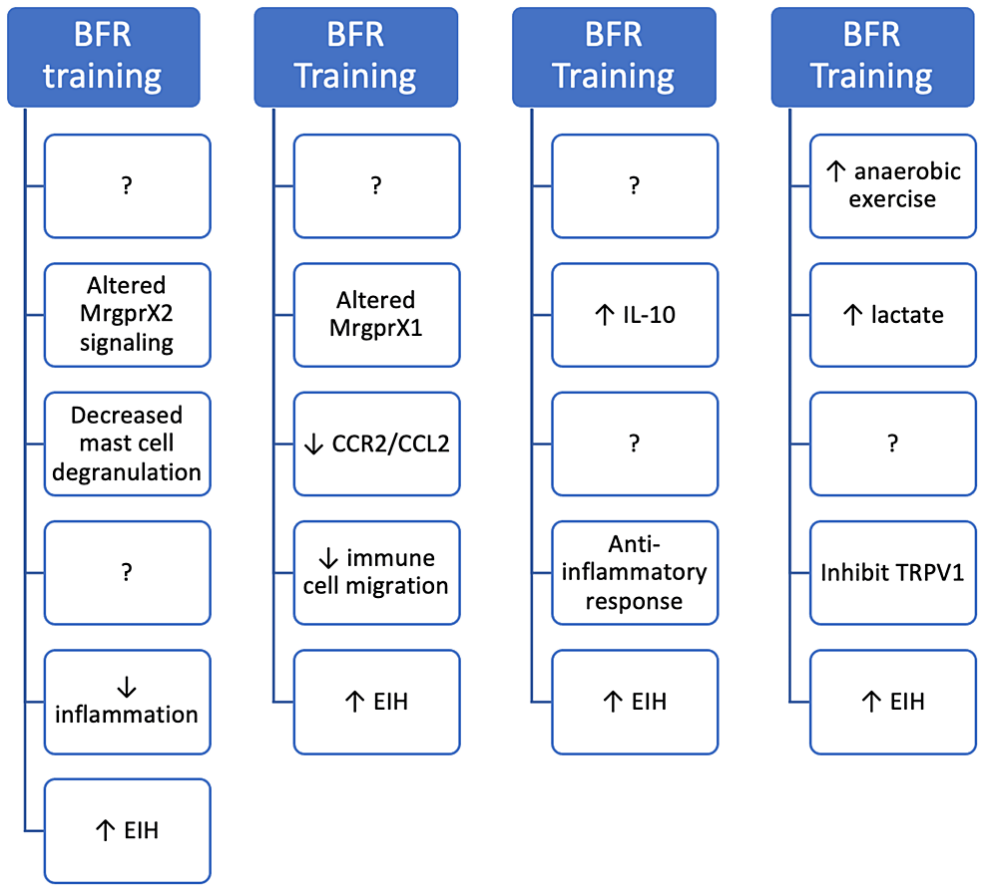
Potential mechanisms for local analgesia. There are several possible mechanisms for the local analgesia experienced following BFR training. EIH is a well-known phenomenon, but to date, there is no explanation for why BFR augments these effects. It is possible that MrgprX2 signaling may be reduced or perhaps signaling downstream of mast cells may be altered. Little is known about MrgprX1 in connection to BFR; blocking this pathway may reduce inflammation. Altered signaling may be in the form of Mrgpr receptor downregulation or inhibition of the binding site. IL-10 is anti-inflammatory, and BFR has been shown to increase IL-10, which could potentially reduce inflammation-mediated nociception. It is unknown if IL-10 concentrations are elevated in regions dense with resident mast cells. Metabolites build up during exercise, and in some instances, lactate may inhibit pain. What is unknown is if there is a correlation between BFR training and exercise metabolite (ATP, lactate) build up after 24 hours. Question marks represent unknowns within the hypothetical pathway [18-35]. Image credit: Giovanni A. Cervini. BFR, blood flow restriction; EIH, exercise-induced hypoalgesia; Mrgpr, Mas-related G-protein-coupled receptor; IL, interleukin; ATP, adenosine triphosphate

Upon stimulation, mast cells will degranulate contributing to pain and inflammation as well as the recruitment of additional immune cells. Immune cell recruitment further elevates the level of inflammation [18]. Inflammation is a common source of pain following exercise, which suggests perhaps a local mechanism may, in fact, be linked to an immune-related response. The explanation for why BFR training feeds into this pathway could be simple as shown in Figure 1. The Mas-related G-protein-coupled receptors (Mrgprs) are densely found in peripheral sensory neurons and play a large role in somatosensation, particularly pain and itch. Colocalized with peripheral nerves are resident mast cells in the skin which can trigger the MrgprX2 variant [19]. The ortholog of the human protein MrgprX2 is the mouse MrgprB2; this receptor has been shown in knockout experiments to be a significant component in postoperative pain and inflammation [20]. MrgprB2 specifically is localized in connective tissue which may coincide with greater local versus systemic hypoalgesia induced by BFR.

With a reduced blood flow to the active limb, immune cell recruitment downstream of the mast cells may become depressed due to lack of flow of recruitable factors following vessel constriction. Downregulating the pro-inflammatory cytokine IL-6 could also be a source of reduced immune cell recruitment. One study examining thigh muscle adaption following ACL rupture and BFR training suggested otherwise since it was found that BFR training and standard exercise had similar IL-6 and IL-6 receptor levels [21]. Reduced blood flow would not likely affect resident mast cell concentration as these are already present in the connective tissue and would not need the vasculature for transport. Also, it is possible that the reduced blood flow is dampening the ability for the endogenous agonists of MrgprX2, such as basic secretagogues or inflammatory peptides, to reach their target location in the connective tissue [19].

This finds some standing in that recent BFR training experiments showed EIH within the knee joint, which is densely packed with connective tissue, and not in the surrounding muscle tissue [22]. However, the pain within the muscle may simply be due to discomfort from the tourniquet. To our knowledge, no research has investigated whether BFR training and EIH are linked to a decrease in Mrgpr signaling, making this a prime target for future study. Despite this lack of focus, there are some intriguing studies which have shown connections between pain and an inflammatory-immune response not pertaining to BFR and exercise specifically, which are detailed in Figure 1.

CCR2/CCL2 Pathway

Another pathway connected to Mrgpr signaling is the chemokine receptor 2/chemokine ligand pathway (CCR2/CCL2). A strong activator of MrgprX1 is bovine adrenal medulla 22 (BAM22), which is a cleavage product of pro-enkephalin A derived from bovine adrenal glands. Unsurprisingly, in addition to MrgprX1, the opioid byproduct BAM22 is also capable of stimulating opioid receptors. MrgprX1 has been shown to centrally inhibit nociceptive responses making it a subject of intense study for its clinical significance in pain management [19]. However, some studies have also demonstrated MrgprX1, upon stimulation, induces the upregulation of CCR2. CCR2 upregulation, mediated by nuclear factors of activated T-cells (NFAT) [23], promotes CCL2 production which ultimately leads to neuropathic pain specifically in the dorsal root ganglion (DRG) [24].

More recent work focused on the inhibition of pain via MrgprX1 signaling, which may seem contradictory to the aforementioned study [24]. However, it is possible that this is a result of autoregulation by CCR2. CCR2 is responsible for CCL2 clearance, so to prevent physiologic excess, CCR2 will trigger the degradation of CCL2 via migrating immune cells [25]. This may be a promising avenue of study given the findings of Blanks et al. [26] who found that with high-intensity exercise CCR2 was downregulated. A decrease in neuropathic pain markers may be the significant alteration needed for the favoring of MrgprX1-mediated inhibition of pain in the DRG. If this is the case, then it may be contributing to the local analgesic effects seen following BFR training. EIH has also been studied in the context of anti-inflammatory cytokines.

Cytokine Modification

IL-10 is an anti-inflammatory cytokine when present in large concentrations [27]. There have been studies done correlating increases in IL-10 protein and mRNA levels within serum and the nervous system following exercise [27,28]. This points to the potential protective nature of IL-10 as it inhibits IgE, which is pro-inflammatory in nature. As IgE induces degranulation of mast cells, there is a potential correlation between IL-10 and the possible inhibition of pro-inflammatory mast cell degranulation. By inhibiting mast cell degranulation, muscle fibers may be spared from inflammation and, therefore, able to induce hypoalgesic effects in the individual’s muscle. This would be outside the Mrgpr pathway as the MrgprX2-induced mast cell degranulation is IgE-independent [18]. BFR training significantly upregulates the production of IL-10 in comparison to standard training groups post 24 hours [29]. The temporal aspect of these findings is most interesting, given our focus of this review. As it was stated earlier, Hughes and Patterson [10] demonstrated EIH after 24 hours in the BFR training groups.

As stated previously, IL-10 concentrations have been shown to increase during exercise and the cytokine has a known anti-inflammatory effect, inhibiting the action of inflammatory mediators such as macrophages and dendritic cells. Considering this, future study investigating the role IL-10 plays following BFR training is appealing. This may be conducted by understanding the findings of Evangelista et al. [29] more clearly through quantification and comparison of the IL-10 levels and PPT at similar time intervals. Furthermore, it would be beneficial to visualize IL-10 localization within regions dense with resident mast cells. If mast cells are, in fact, significantly inhibited following BFR training, the next step in discovery would be to understand why IL-10 is upregulated. Normally, in response to exercise induced muscle damage, the body responds by vasodilating to increase cytokine migration as well as nutrient and oxygen delivery to damaged tissues. With vessel occlusion by use of tourniquet, this mechanism may be reduced and therefore decrease the pain response associated with inflammation.

Exercise Metabolites

Despite well-known research that exercise metabolites such as ATP, H+, and lactate cause the sensation of fatigue and pain [30,31], lactate has been shown in vitro to inhibit pain response via the transient receptor potential vanilloid 1 (TRPV1) signal pathway [32]. Furthermore, lactate alone does not appear to induce the sensation of pain, but only when in combination with specific concentrations of ATP and H+ [33]. The findings in the study by Hughes and Patterson [10] suggest not only that there is not only a systemic hypoalgesic effect with BFR training but also that the hypoalgesic effect is increased compared to normal blood flow exercise and that this effect endures past the 24-hour mark. The presence of hypoalgesia past 24 hours, as well as the normal levels of endocannabinoids and opioids, suggests that an additional pathway is present outside of the standard endocannabinoid and opioid pathways previously mentioned.

**Figure 2:**
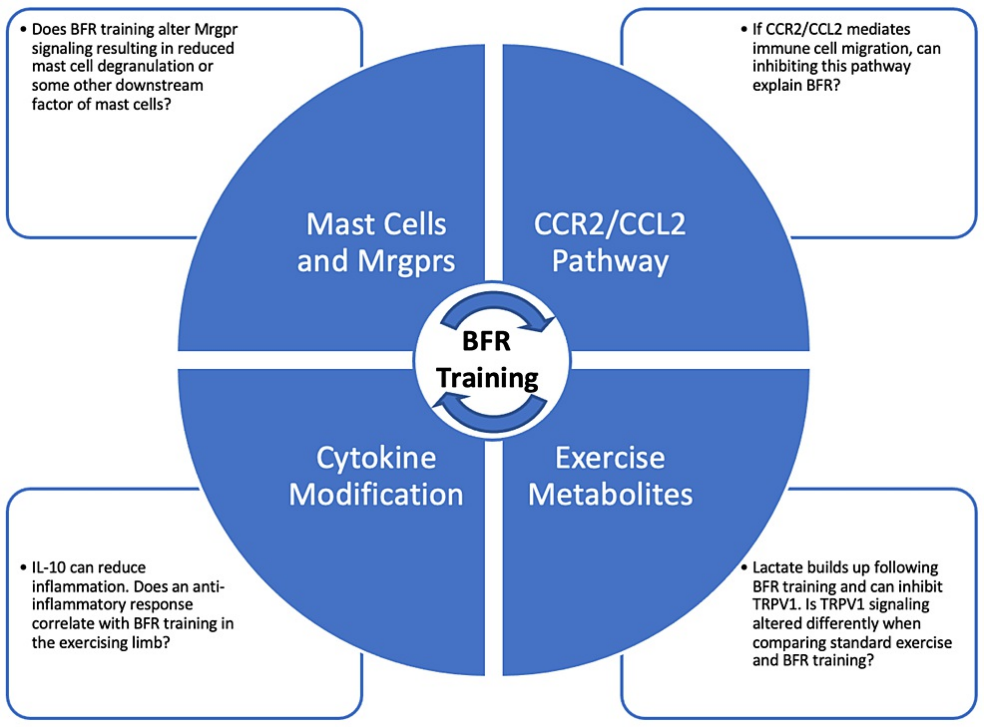
Future directions. There are several questions we recommend need further investigation to determine why BFR augments EIH. It is possible that there may be a combination of these mechanisms. If this is the case, then any experiment investigating these factors will be complex and the results perhaps difficult to interpret. Image credit: Giovanni A. Cervini. BFR, blood flow restriction; EIH, exercise-induced hypoalgesia; Mrgpr, Mas-related G-protein-coupled receptor; IL, interleukin; CCR2/CCL2, chemokine receptor 2/chemokine ligand pathway; TRPV1, transient receptor potential vanilloid 1

There are several questions left unanswered for the previously mentioned pathways (Figure 2). To further understand the role lactate plays in the local sensation of pain, it would be beneficial to measure ATP, pH, and lactate at the post 24-hour mark. It is already known that BFR training significantly increases lactate and reduces the pH in comparison to standard exercise [34]. Some research has suggested that BFR training provides a long-term effect of improving pH and lactate regulation in the exercising muscle [35]. While being both a significant and important finding, one limitation of this study is that measurements were taken after six weeks and do not give us a complete understanding of what happens immediately after exercise. Thus, it would be beneficial to evaluate the transcriptional level of H+ and lactate transport proteins at the 24-hour mark to better relate the findings from Christiansen et al. [35] to Hughes and Patterson [10].

Beyond Sports

Although athletes are the primary focus of this review, it is not difficult to see the potential of BFR training for other patients outside of the sports realm. The postoperative patient is a prime candidate for BFR training as muscle atrophy is often a consequence of inactivity. With atrophy comes a poorer outcome in return of physical function [36]. The principle works the same for a postoperative patient as it would for a recovering athlete: a patient gains the EIH benefit of diminished pain and energy expenditure as well as a reduced physical load, enabling a more efficient recovery process. Even populations with chronic pain disorders may be able to benefit from EIH if properly implemented. Fibromyalgia syndrome (FMS) is a disease of unknown origin that is characterized by diffuse pain, anxiety, and fatigue; a well-established treatment of FMS is exercise therapy, which has been shown to reduce symptom severity and improve quality of life [37]. Given that no study has yet to be conducted using BFR training as a method of treatment for FMS, there is a potential opportunity for advancement in the control of this debilitating disease.

## Conclusions

In summary, BFR training is a unique and effective tool for the sports medicine professional. Normally, injured patients suffer through the painful process of rehabilitation, recovery, and adaptation to their injuries. However, by using BFR, the road to recovery impose a lesser burden on the patient. While current research has pointed to the potential of BFR training and its ability to enhance hypoalgesia achieved through exercise, future study is needed to determine the clinical significance to sports medicine patients. In addition to establishing the effectiveness of this mechanism for the short term, further inquiries need to be done on the long-term outcome of these patients. While an earlier return to play is, in many ways, good for the short term, long-term outcomes related to injury have yet to be established when using BFR and EIH. Through further research, the complex mechanisms underlying EIH and BFR training can be better understood to modify and improve the current training regimens used. Specifically, Mrgprs, CCR2/CCL2, IL-10, and lactate show promising opportunities moving forward for the future of injury rehabilitation. This has the potential to not only help athletes recover from injuries but also benefit other patient populations within medicine. Moving forward, we hope that more information will be uncovered with time to better implement exercise and BFR training.
